# Is right-sided ligamentum teres hepatis always accompanied by left-sided gallbladder? Case reports and literature review

**DOI:** 10.1007/s13244-018-0671-9

**Published:** 2018-11-19

**Authors:** Hsuan-Yin Lin, Rheun-Chuan Lee

**Affiliations:** 1 Department of Radiology, Taipei Veterans General Hospital, No. 201, Sec. 2, Shipai Rd., Beitou District, Taipei, 112 Taiwan Republic of China; 2 School of Medicine, National Yang-Ming University, Taipei, Taiwan Republic of China

**Keywords:** Right-sided ligamentum teres (RSLT) hepatis, Left-sided gallbladder, Maximum intensity projection (MIP), Magnetic resonance cholangiopancreatography (MRCP)

## Abstract

**Abstract:**

Right-sided ligamentum teres (RSLT) hepatis is a rare anatomical variant in which the fetal umbilical vein is connected to the right paramedian trunk of the portal vein. Despite its rarity, it is crucial for surgeons and intervention specialists because of its frequent association with intrahepatic vascular and biliary anomalies. Inattention to these anomalies before intervention, especially living-donor liver transplantation, can have life-threatening consequences. The relationship between gallbladder location and RSLT is still controversial, with RSLT regarded as one of the critical features of left-sided gallbladder in most studies. According to these hypotheses, once RSLT is present, left-sided gallbladder must be found as well. Here, we report three cases in which RSLT was associated with intrahepatic portal vein anomalies. In one case, the gallbladder was left-sided, but in the other two cases, it had a normal cholecystic axis to the right of the umbilical fissure. Therefore, the relationship between RSLT and gallbladder location may require redefinition, and surgeons should be aware of vascular anomalies once RSLT has been detected, even in the absence of left-sided gallbladder or biliary anomalies.

**Teaching Points:**

*• Right-sided ligamentum teres (RSLT) hepatis is a rare anatomical variant, which is frequently associated with intrahepatic vascular and biliary anomalies. Previous studies had discussed the vascular anomalies in livers with RSLT.*

*• However, no predictable correlation exists between portal vein anomalies and anomalous biliary confluences in patients with RSLT. Moreover, we found that RSLT does not always coexist with left-sided gallbladder.*

*• Unawareness of these vascular and biliary anomalies in liver with RSLT before intervention can have life-threatening consequences.*

*• Thus, the vascular and biliary variations should be surveyed in multimodality imaging studies such as dynamic CT, 3D magnetic resonance cholangiopancreatography, or digital subtraction angiography once the RSLT is detected before intervention.*

## Case 1

**Fig. 1:**
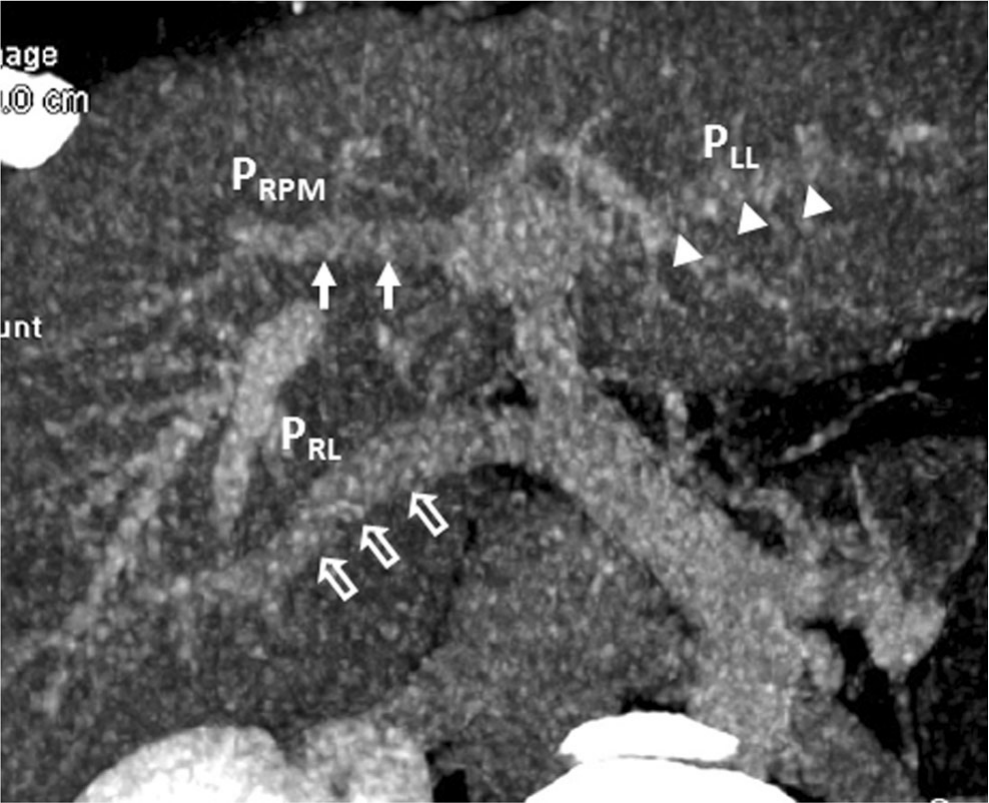
Contrast-enhanced computed tomography (CT) with maximum intensity projection (MIP) reconstruction revealing portal vein ramification of a Shindoh’s independent right lateral type

**Fig. 2:**
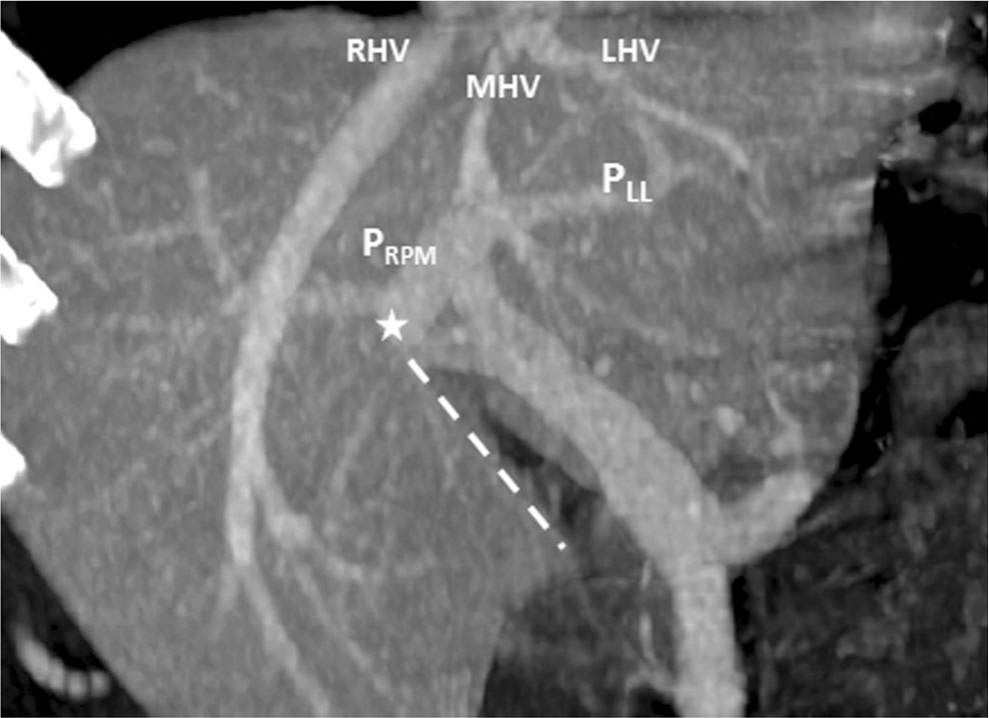
Contrast-enhanced CT scan revealing the right paramedian portal pedicle (P_RPM_) forming the right umbilical portion of the portal vein (*star*) and joining the right-sided ligamentum teres (RSLT) (*dotted line*), with the middle hepatic vein (MHV) running to the left of the umbilical portion and RSLT

**Fig. 3:**
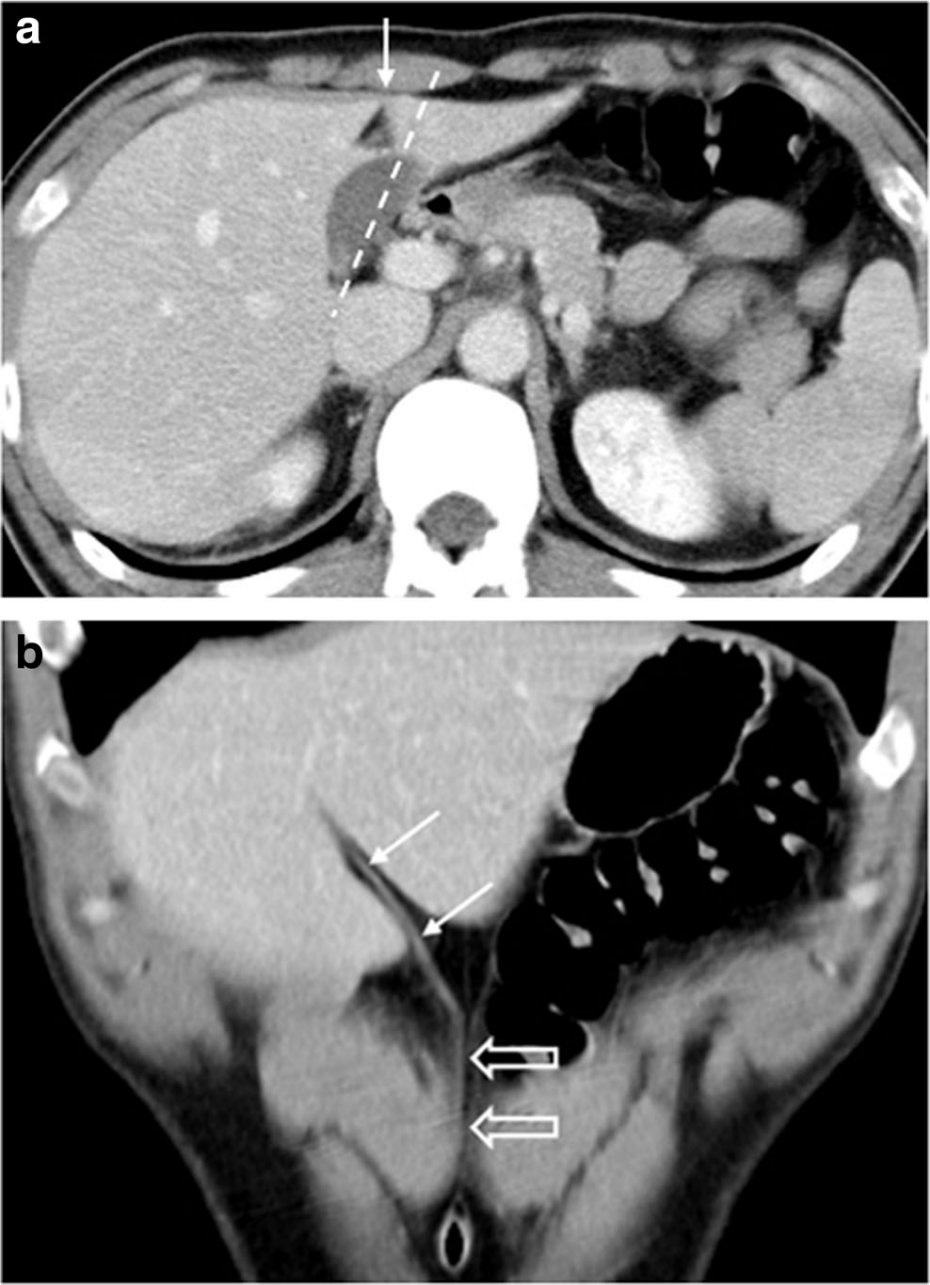
**a** Contrast-enhanced CT scan revealing the gallbladder’s cholecystic axis (*dotted line*) left of the umbilical fissure and RSLT (*arrow*). **b** The umbilical ligament (*thick arrows*) joined with the RSLT (*thin arrows*) is shown

A 37-year-old man underwent contrast-enhanced computed tomography (CT) and magnetic resonance imaging (MRI) to evaluate hepatic nodules incidentally detected in ultrasonography. The CT and MRI results revealed portal vein ramification of a Shindoh’s independent right lateral type [1]. The first branch of the portal vein issued into the right lateral branch and then formed the trunk of the left portal vein and right paramedian portal veins. The right paramedian portal pedicle then formed the right umbilical segment of the portal vein and joined with the right-sided ligamentum teres (RSLT) (Figs. 1 and 2). The middle hepatic vein (MHV) was located to the left of the RSLT, which is one of the characteristic imaging features of RSLT [1] (Fig. 2). The gallbladder lay with its cholecystic axis to the left of the umbilical fissure (Fig. 3a, b). The hepatic nodules were radiologically diagnosed as hemangiomas.

## Case 2

**Fig. 4:**
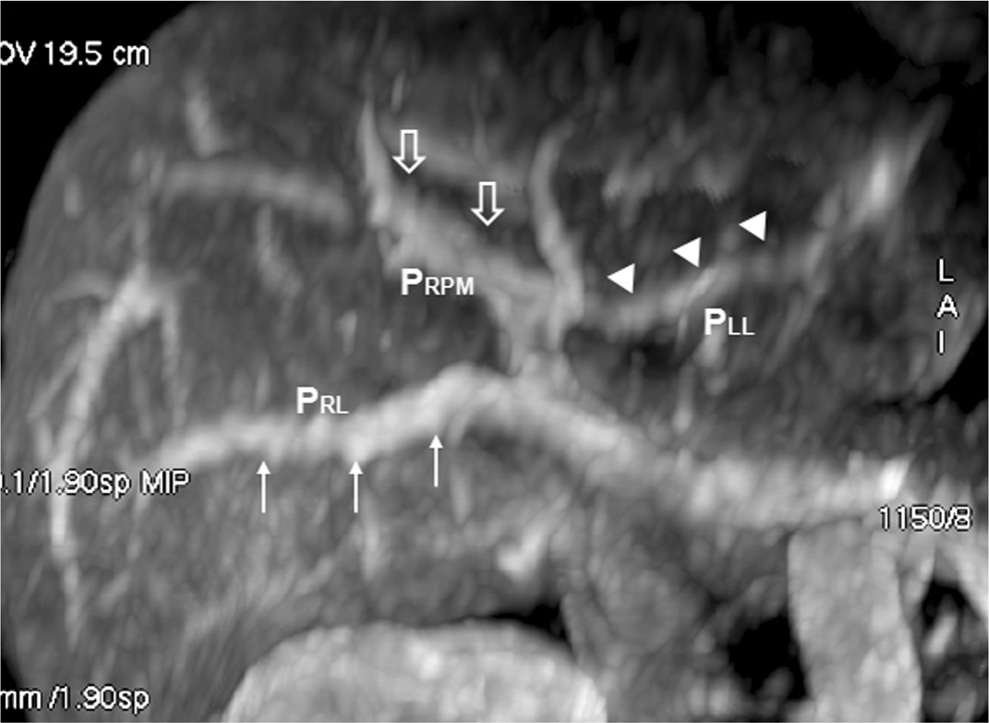
Contrast-enhanced T1 fat-sat magnetic resonance imaging (MRI) with maximum intensity projection (MIP) reconstruction revealing portal vein ramification of a Shindoh’s independent right lateral type

**Fig. 5:**
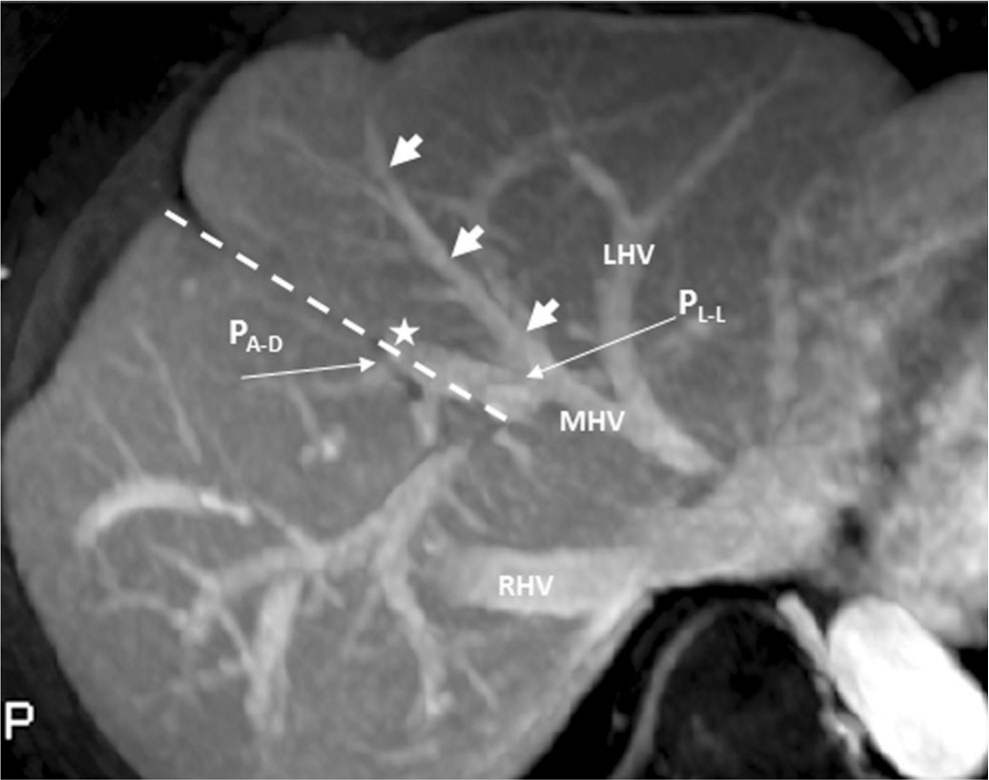
Contrast-enhanced MRI with MIP reconstruction revealing the MHV running to the left (*short arrows*) of the umbilical portion (*star*) and RSLT (*dotted line*), with the umbilical portion (*asterisk*) tilting to the right and joining with the RSLT. The diverging point of the dorsal branch of the right anterior portal vein (P_A–D_) is distal to that of the left lateral portal vein (P_LL_)

**Fig. 6:**
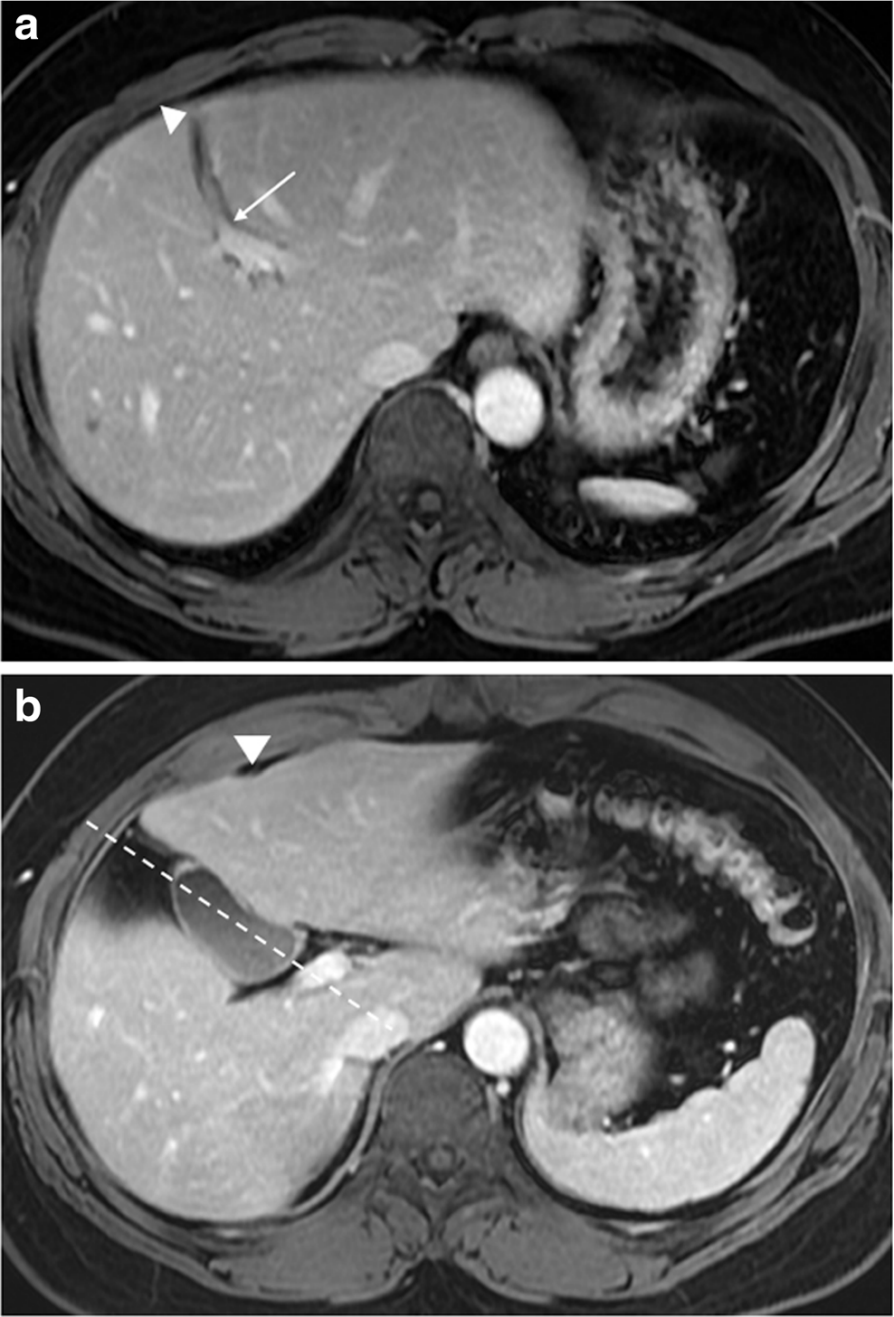
**a** Contrast-enhanced T1 fat-sat MRI revealing the right paramedian portal vein forming the umbilical portion (*arrow*) of the portal vein and joining with the RSLT (*arrowhead*). **b** The gallbladder has a normal cholecystic axis (*dotted line*) to the right of the umbilical fissure (*arrowhead*)

**Fig. 7:**
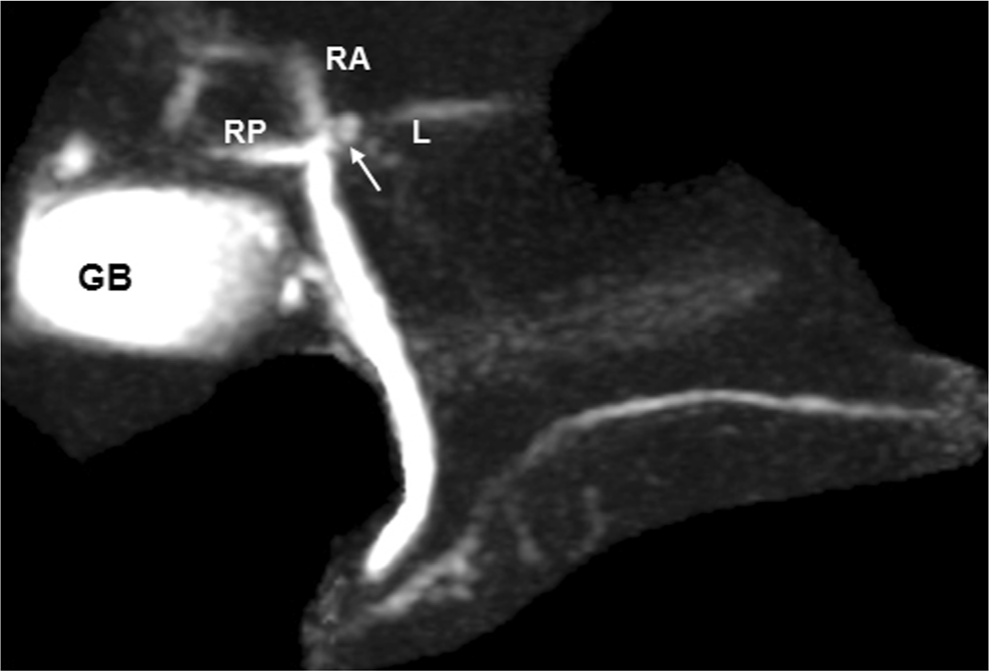
Magnetic resonance cholangiopancreatography (MRCP) revealing the right anterior hepatic duct (*RA*) in a confluence (*arrow*) with the left hepatic duct (*L*) before draining into the common bile duct (CBD). The right posterior hepatic duct (*RP*) drains into the CBD directly. The gallbladder (*GB*) is in its normal right-sided position

A 42-year-old man with choroidal malignant melanoma underwent a regular MRI examination for possible distant metastasis. The MRI revealed portal vein ramification of a Shindoh’s independent right lateral type [1], with the umbilical portion of the portal vein tilting to the right and joining with the RSLT and the MHV running to the left of the RSLT (Figs. 4 and 5). The diverging point of the dorsal branch of the right anterior portal vein (P_A–D_) was distal to that of the left lateral portal vein (P_LL_), which is the opposite of normal anatomy and one of the axial imaging features described by Yamashita et al. [2] for identifying RSLT (Fig. 5). The gallbladder had a normal cholecystic axis to the right of the umbilical fissure (Fig. 6a, b). Magnetic resonance cholangiopancreatography (MRCP) revealed right anterior hepatic duct confluence with the left hepatic duct before draining into the common bile duct (CBD), whereas the right posterior hepatic duct drained into the CBD directly, just following the portal ramification. The gallbladder was in its normal right-sided position (Fig. 7).

## Case 3

**Fig. 8:**
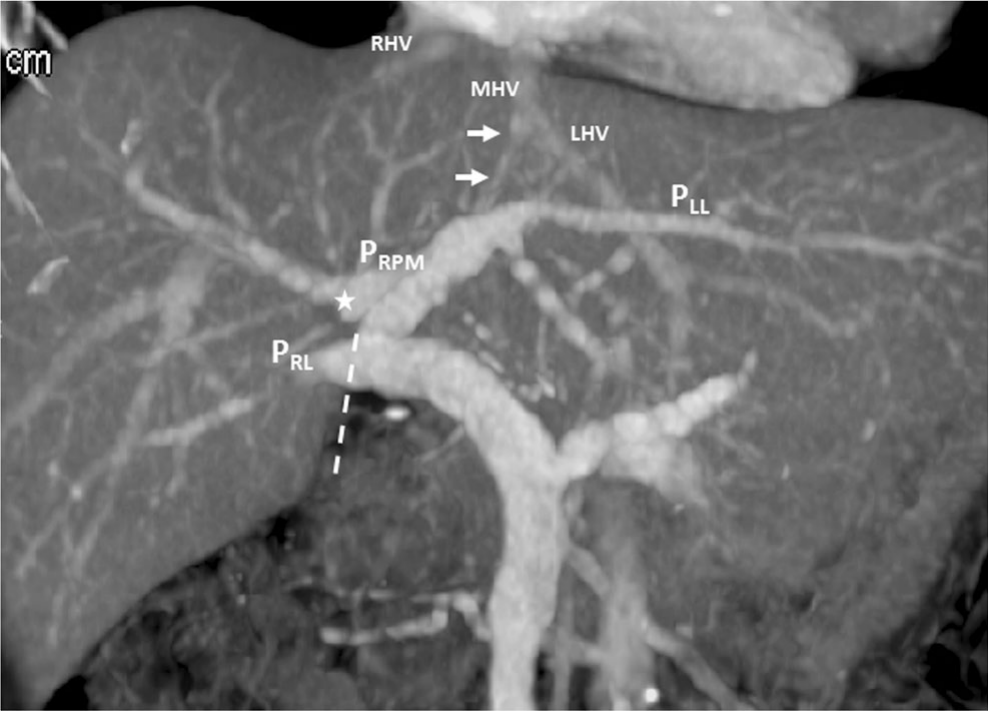
Contrast-enhanced CT with MIP reconstruction revealing portal vein ramification of a Shindoh’s independent right lateral type, with the umbilical portion (*asterisk*) of the portal vein tilting to the right and joining with the RSLT (*dotted line*) and the MHV running to the left (*arrows*) of the RSLT

**Fig. 9:**
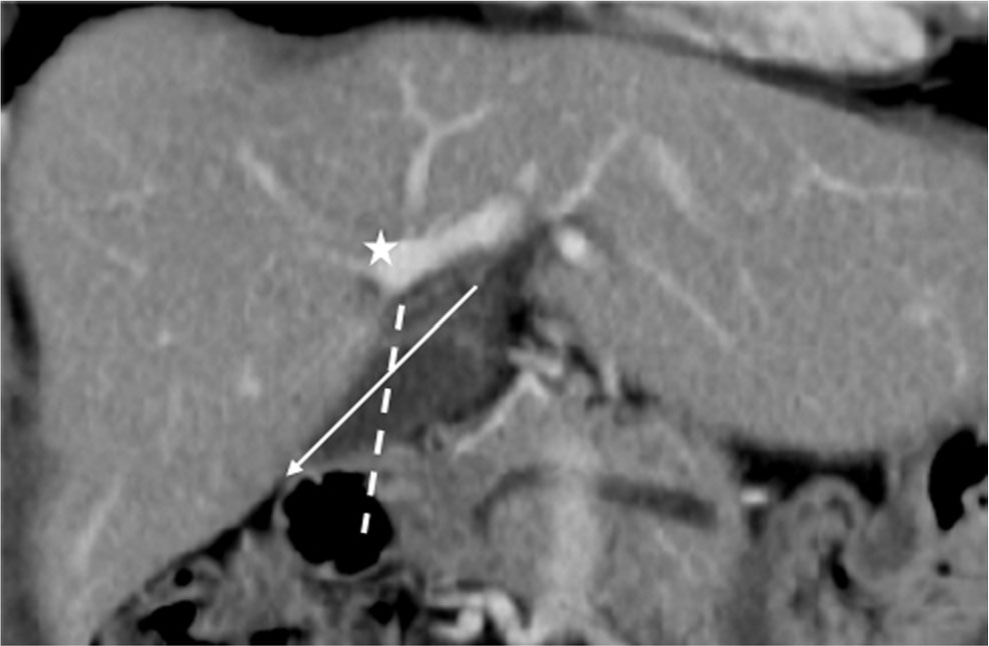
Contrast-enhanced CT revealing the gallbladder with a normal cholecystic axis (*arrow*) to the right of the umbilical fissure (*star*) and RSLT (*dotted line*)

A 72-year-old woman with rectal cancer underwent a regular dynamic contrast-enhanced CT survey for possible distant metastasis. The CT images revealed portal vein ramification of a Shindoh’s independent right lateral type [1], with the umbilical portion of the portal vein tilting to the right and joining with the RSLT and the MHV running to the left of the RSLT (Fig. 8). The gallbladder had a normal cholecystic axis to the right of the umbilical fissure (Fig. 9).

## Discussion

**Fig. 10:**
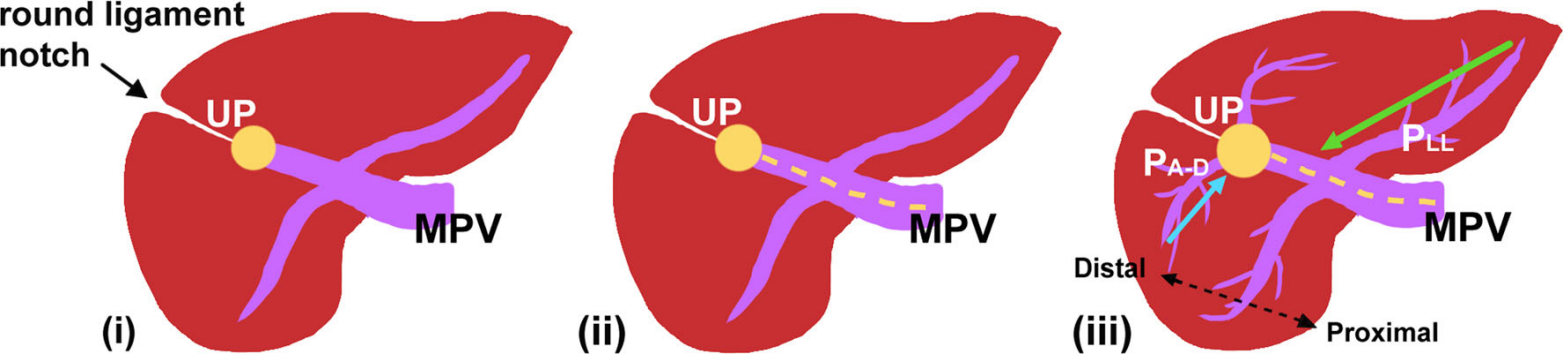
Three-step method for detection right-sided ligamentum teres (RSLT) hepatis in axial images established by Yamashita et al. [2]. (i) The first step: identify the connection of the round ligament (or the round ligament notch) to the umbilical portion of the portal vein (UP, *yellow circle*). (ii) The second step: set an axis (*dotted line*) on the portal vein from the main portal vein (MPV) to the UP. (iii) The third step: identify the diverging points of the dorsal branch of the right anterior portal segment (P_A–D_, *blue arrow*) and the left lateral portal segment (P_LL_, *green arrow*). The diverging point of P_A–D_ is distal to that of P_LL_ in RSLT liver and proximal in normal liver

**Fig. 11:**
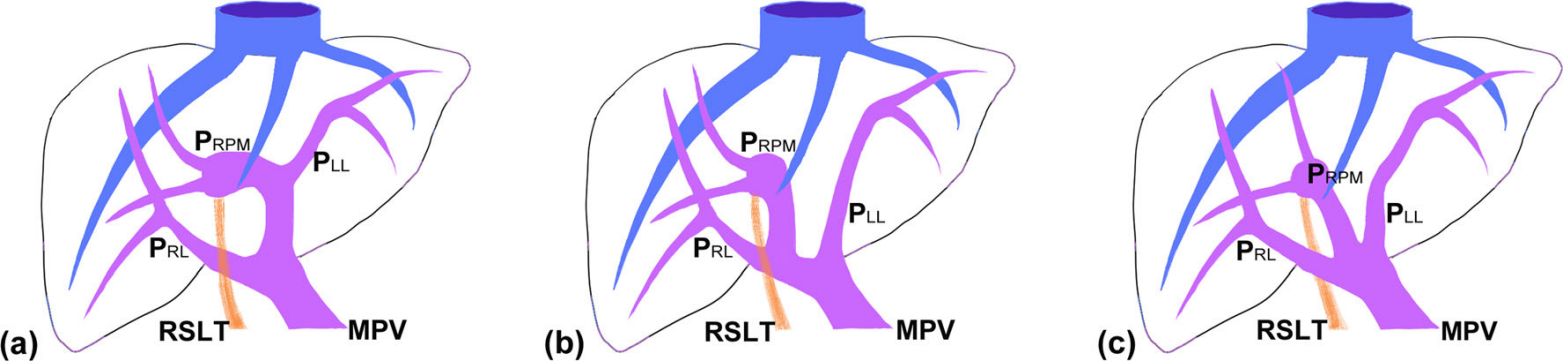
Schematic representation of the intrahepatic portal venous system anomalies classified by Shindoh et al. [5]. **a** The independent right lateral type: the right lateral portal pedicle (P_RL_) origins from the MPV independently and the right paramedian portal pedicle (P_RPM_) shares the common trunk with the left lateral portal vein(P_LL_). **b** Bifurcation type: the MPV bifurcating into the right and left portal trunks first and the P_RL_ origins from the right portal trunk as P_RPM_. **c** Trifurcation type: the MPV divided into three branches of P_RL_, P_RPM_, and P_LL_ immediately

RSLT and the right umbilical portion of the portal vein were first reported in 1986 by Matsumoto [3], with a reported prevalence of 0.1–1.2% in the adult population [4]. Matsumoto assumed that persistence of the right umbilical vein rather than the left one could result in misconnection of the ligamentum teres on the right side [3]. This hypothesis is supported objectively by the vascular territories, the segmental volumes reported by Shindoh et al. [1, 5], and neonatology ultrasound findings [6]. A three-step method for the detection of RSLT in axial images was established by Yamashita et al. (Fig.10) [2] on the basis of the diverging points of the dorsal branch of the right anterior portal vein (P_A–D_) and the lateral segmental portal (P_LL_) vein: the diverging point of P_A–D_ is distal to that of P_LL_ in an RSLT liver and proximal in a normal one. The portal venous ramification patterns in RSLT livers were classified by Shindoh et al. [1] into three types according to the origin of the right lateral portal pedicle, namely, the bifurcation, trifurcation, and independent right lateral types, the third type being the most common pattern in RSLT livers (Fig. 11). Shindoh et al. believed that, when RSLT is present, the gallbladder must be found in a reversed position and the MHV must lie to the left of the RSLT [1, 5]. The RSLT presented in the reports are recognized by the notch of round ligament (or notch of ligamentum teres) directed connected with the umbilical segment of the portal vein that is derived from right portal branches and the MHV was located to the left of the RSLT, following the definitions and consistent with the findings elaborated by Shindoh et al. [1].

A left-sided gallbladder without situs inversus was first described by Hochstetter in 1886 [7], and a multicenter series of laparoscopic cholecystectomies has indicated a prevalence of 0.3% [7–9]. There has been much debate and controversy about the true definition of left-sided gallbladders [10, 11] and the relationship between gallbladder position and ligamentum teres. The simple definition was that of a gallbladder located on the undersurface of the left lobe, with only two theories for its development (i.e., aberrant drawing of the pars cystica toward the left or accessory gallbladder from the left hepatic duct with regression of the main gallbladder), until Nagai et al. cautioned that some reports of left-sided gallbladders may have been erroneous [10, 11]. It was proposed that, rather than the gallbladder, it was the ligamentum teres whose unusual location caused the anatomical variation. This was because, according to the limited explanation of the earlier hypothesis, a left-sided gallbladder must be located to the left of not only the round ligament but also the MHV, whereas the round ligament itself should originate from the left portal vein.

The following four explanations have been offered for the development of a left-sided gallbladder without situs inversus [9]:

1. The gallbladder bud migrates to the left lobe (the portal vein, biliary tree, and hepatic artery should be in their normal position and classified as an ectopic gallbladder).
2. The gallbladder develops directly from the left hepatic duct, with failed development of the normal structure on the right side (cystic duct from the left hepatic duct).
3. The left umbilical vein disappears, whereas the right umbilical vein partly remains, with its peripheral and central portions developing into the ligamentum teres and ligamentum venosum, respectively. According to this (Matsumoto’s) hypothesis, the right umbilical portion should lie to the right of the gallbladder bed.
4. The ligamentum teres simply deviates to the right.

These hypotheses seek to explain the relationship between RSLT, intrahepatic portal vein anomalies, and left-sided gallbladder. All of them [1, 5, 9] imply that, once RSLT is present, a left-sided gallbladder must be found as well. However, in the cases reported by Yamashita et al. [2], RSLT could be present with the gallbladder located just beneath, to the left, or to the right of the round ligament. We have presented another two cases where RSLT was present without a left-sided gallbladder. The 3D MRCP and MIP reconstruction used in our cases provide objective information about portal flow and biliary confluence in RSLT livers.

RSLT is frequently accompanied by intrahepatic vascular anomalies and anomalous biliary confluences [1, 2, 5]. However, no predictable correlation exists between portal vein anomalies and anomalous biliary confluences in patients with RSLT [5], despite the fact that vascular anomalies in RSLT livers have been thoroughly discussed and classified [1, 2, 5]. Moreover, we found that RSLT does not always coexist with left-sided gallbladder. Consequently, the vasculature and biliary structure should be surveyed carefully in preoperative imaging studies when RSLT is detected, even in the absence of left-sided gallbladder. Inattention to such anomalies before intervention can have life-threatening consequences. Because independent ramification of the right lateral portal pedicle is the most common RSLT type, ligation of the left trunk of the portal vein during hepatobiliary surgery will disrupt portal flow in the left two-thirds of the entire liver if the common trunk of the left portal vein and right paramedian pedicle is misinterpreted as the left portal vein [12]. Serious biliary complications during major hepatobiliary interventions in patients with RSLT have also been reported [13, 14]. The relationship between RSLT and biliary confluences may require further investigation and a redefinition. With the increasing popularity of 3D MRCP, an extremely low-risk examination that requires no contrast medium injection and only a relatively short examination time, a better understanding of biliary confluences in RSLT livers can be achieved.
